# Synergy between dinotefuran and fipronil against the cat flea (*Ctenocephalides felis*): improved onset of action and residual speed of kill in adult cats

**DOI:** 10.1186/s13071-017-2272-8

**Published:** 2017-07-19

**Authors:** Romain Delcombel, Hamadi Karembe, Bakela Nare, Audrey Burton, Julian Liebenberg, Josephus Fourie, Marie Varloud

**Affiliations:** 1 Ceva Santé Animale, 10 avenue de la Ballastière, 33500 Libourne, France; 2 Clinvet International (Pty) Ltd, Uitzich Road, Bainsvlei, Bloemfontein, South Africa; 3 Avista Pharma Solutions, 3501-C TriCenter Blvd, Durham, NC 27713 USA

**Keywords:** Dinotefuran, Fipronil, Synergy, Efficacy, In vitro, In vivo, Fleas, *Ctenocephalides felis*

## Abstract

**Background:**

The cat flea, *Ctenocephalides felis felis* (*C. felis*), is a cosmopolitan hematophagous ectoparasite, and is considered to be the most prevalent flea species in both Europe and the USA. Clinical signs frequently associated with flea bites include pruritus, dermatitis and in severe cases even pyodermatitis and alopecia. *Ctenocephalides felis* is also a vector for several pathogens and is an intermediate host for the cestode *Dipylidium caninum.* Treatment of cats with a fast-acting pulicide, that is persistently effective in protecting the animal against re-infestation, is therefore imperative to their health. In addition, a rapid onset of activity (“speed of kill”) may also reduce the risks of disease transmission and flea allergic dermatitis. The aim of this study was to evaluate the in vitro insecticidal activity and potential synergism between dinotefuran and fipronil against *C. felis*. A further aim was to evaluate the onset of activity and residual speed of kill of the combination in vivo on cats artificially infested with *C. felis.*

**Methods:**

In the first study, the insecticidal activity of dinotefuran and fipronil separately and dinotefuran/fipronil (DF) in combination, at a fixed ratio (2:1), was evaluated using an in vitro coated-vial bioassay. In the second study, the onset of activity against existing flea infestations and residual speed of kill of DF against artificial flea infestations on cats was assessed in vivo. Onset of activity against existing flea infestations was assessed in terms of knock-down effect within 2 h post-treatment and onset of speed of kill assessed at 3 h, 6 h and 12 h post-treatment. Residual speed of kill was evaluated 6 h and 48 h after infestation, over a period of six weeks post-treatment.

**Results:**

In vitro results revealed that the DF combination was synergistic and more potent against fleas than either compound alone. The combination also proved effective when tested in vivo*.* Efficacy was > 97% [geometric mean (GM) and arithmetic mean (AM)] at 3 h after treatment, and ≥ 99.8% (GM and AM) at 6 h and 12 h post-treatment. At 6 h after flea re-infestations, the efficacy of DF remained ≥ 90.8% (GM and AM) for up to 28 days, and at 42 days post-treatment persistent efficacy was still ≥ 54.3% (GM and AM). At 48 h after flea re-infestations, DF remained almost fully effective for up to 28 days, with efficacies ≥ 99.4% (GM and AM) and was persistently ≥ 93.0% (GM and AM) effective for up to 42 days post-treatment.

**Conclusions:**

The combination of dinotefuran and fipronil in a single formulation exhibited strong synergistic insecticidal activity against *C. felis* in vitro, and also proved effective on artificially infested cats. This activity had a rapid onset that persisted for 6 weeks against re-infestations of *C. felis* on cats. The rapid curative insecticidal effect was observed as early as 3 h after treatment, and as early as 6 h after re-infestations for up to 6 weeks post-treatment. The insecticidal activity profile of DF makes it an optimal candidate for the protection of cats against flea infestations, and possibly also associated diseases.

## Background

The cat flea, *Ctenocephalides felis felis* (*C. felis*) is a hematophagous ectoparasite that infests cats and other domestic animals worldwide [1]. Both outdoor and indoor domestic animals are affected. A recent epidemiological study conducted in several European countries, indicated that 15.5% of cats were infested with fleas [2]. Moreover, *C. felis* was the most prevalent flea species in this study. Similarly, *C. felis* has recently been reported to be the most prevalent flea species in the USA [3]. In this epidemiological survey conducted by Blagburn and collaborators in 2016, 96% of the fleas collected countrywide were identified as *C. felis*.

Fleas are highly efficient in acquiring a host by leaping from their immediate environment onto such an animal [4]. In addition, when more than one animal is accommodated in a household, an infested host can transmit live and prolific fleas to its congeners, and this inter-host transfer can occur within an hour after contact [5]. Immediately after a host has been acquired, fleas start feeding and continue to take numerous blood meals daily [6–9].

Consequently, symptoms associated with flea infestations can soon be observed. For instance, allergenic proteins contained in *C. felis* saliva, may result in immediate hypersensitivity resulting in flea allergic dermatitis (FAD) [10]. Cats with FAD suffer from intense discomfort caused by severe pruritus and dermatitis characterized by excoriation, scaling, crusting, miliary lesions and papules. If untreated, the disease can lead to pyodermitis and alopecia. Although the threshold in unknown, a small number of flea bites are expected to induce a resurgence of the symptoms in cats already sensitized to flea allergens [11]. The detrimental effects of flea infestations are, however, not limited to sensitized animals, but can be responsible for skin irritation in non-flea allergen sensitized hosts, resulting in intense grooming and itching, and can even induce anemia in susceptible cats [11].

*Ctenocephalides felis* is also a vector of several pathogens, namely viruses such as feline leukemia virus [12], bacteria such as *Rickettsia felis* [13], *Haemoplasma* species [14] and *Bartonella* species [15]. Fleas also act as intermediate host for intestinal helminths such as the cestode *Dipylidium caninum* [16]. Effective treatment of cats with a fast-acting pulicide, with a persistent efficacy against re-infestation, is therefore imperative to their health. Whilst the efficacy of pulicidal products has generally been evaluated 48 h after treatment against existing flea infestations or re-infestation [17], some of them, especially spot-on formulations, are expected to act much sooner. This property, referred to as speed of kill, represents the rapid onset of activity, thus freeing cats from their fleas. Rapid speed of kill may also reduce the risks of disease transmission and FAD [18].

Dinotefuran is a furanicotinyl insecticide belonging to the third generation of neonicotinoids [19]. It acts specifically on the nervous system of fleas by inhibiting a nicotinic subclass of acetylcholine receptors. Dinotefuran is a contact pulicide and has a rapid speed of kill against fleas, as early as 2 h [20] to 6 h post-treatment on infested cats [21]. Moreover, it has been demonstrated that dinotefuran has a residual efficacy lasting for 30 days, when evaluated against 4 consecutive weekly infestations with fleas [22]. The activity of this compound is, however, limited to insects and no acaricidal activity has been demonstrated, except at high concentrations and after a week of exposure [23, 24]. Fipronil on the other hand, is a proven insecticide and acaricide. This compound acts on insects and acarines by blocking the action of gamma-aminobutyric acid. It also acts by contact and because it accumulates in the skin and sebaceous glands, remains active for at least 4 weeks against fleas and ticks [25].

In order to strengthen and extend insecticidal activity, a combination of active ingredients with different modes of action and potency in a single spot-on formulation has been proposed [26, 27]. Moreover, if compounds in combination are synergistic, the same insecticidal efficacy can be achieved as when they are administered separately, but at lower active ingredient concentrations. This is likely to improve the safety profile of such products. In this study, dinotefuran and fipronil were combined in a single solution, taking advantage of their different mechanisms of action. It was anticipated that this novel combination would provide a more complete topical protection of cats against ectoparasites, a rapid onset of efficacy to alleviate flea bite dermatitis and a long lasting residual speed of kill effect, aiding in the protection of cats against FAD and flea-borne pathogens.

The aim of this study was to evaluate the insecticidal activity and potential synergism between dinotefuran and fipronil against *C. felis* in vitro, when tested separately or in combination, and also to evaluate the onset of activity and residual speed of kill in vivo on artificially infested cats.

## Methods

### Design

#### In vitro insecticidal activity and interaction between dinotefuran and fipronil

The insecticidal activity of dinotefuran, fipronil and dinotefuran/fipronil (DF) in a 2:1 fixed ratio (2:1) was evaluated using an in vitro coated-vial bioassay. Test compounds were dissolved in DMSO to a final stock concentrations. An aliquot was taken from each compound stock and added to an acetone/triton solution to achieve the desired top concentrations for the study. The top concentrations of test compound, individually or in combination, were serially diluted with the same diluent to achieve the desired titration range. Vials were treated with dinotefuran, fipronil, DF or solvent alone. The final DMSO concentration in each vial was less than 0.5%. Vials were capped and allowed to dry for at least 4 h before adding 10 newly emerged (0 to 7 days old) unfed adult fleas in each vial. Flea susceptibility was assessed at 48 h post-exposure by evaluating mortality. Those showing normal movement and/or jumping ability were considered live, and those showing no movement after tapping the vials were scored as dead.

#### In vivo efficacy study on cats

An in vivo efficacy study was designed to assess the onset of activity against existing flea infestations, as well as residual speed of kill of DF (2:1 ratio) against artificial flea infestations on cats. This study was an unblinded, randomized, 3-arms study, comparing the results obtained on 2 groups of cats treated with the DF combination with the untreated control group. Group 1 was untreated, Groups 2 and 3 were treated with DF. Groups 1 and 2 were used to evaluate the onset of activity of DF against existing flea infestations, while Groups 1 and 3 were used to evaluate its residual speed of kill. The study was conducted in accordance with the appropriate European and International guidelines at the time [17, 28].

### Animals, animal housing and environmental monitoring

Adult domestic short hair type cats originating from a purpose-bred colony were used in this study (Tables 1, 2). Before enrollment, cats had not been treated with an acaricide, insecticide or an insect growth regulator for at least 12 weeks, were free of fleas and were dewormed. A total of 34 cats were enrolled in the study of which 24 were included in the experimental phase after an acclimatization period of 7 days.

**Table 1:** Details on age and gender of cats included in the study

Group	Treatment	Age in years			Gender		*n*
		Mean	Min	Max	Female	Male	
1	Untreated	4.0	0.9	9.0	5	3	8
2	DF	5.2	3.9	7.0	6	2	8
3	DF	3.9	1.6	7.6	5	3	8

*Abbreviations*: Min, minimum; Max, maximum; n, number; DF, treated with combination of dinotefuran and fipronil

**Table 2:** Details on body weight and hair lengths of cats included in the study

Group	Treatment	Body weight (kg)			Hair length (mm)		
		Mean	Min	Max	Mean	Min	Max
1	Untreated	3.0	2.1	3.8	17.2	13.0	22.3
2	DF	3.1	2.5	3.7	20.6	16.0	25.3
3	DF	3.5	2.6	4.6	17.8	14.8	21.3

*Abbreviations*: Min, minimum; Max, maximum; DF, treated with combination of dinotefuran and fipronil

Cats were included in the study if they were considered healthy based on a veterinary examination, and if their pre-treatment flea retention was > 60%. The animals were housed individually in a temperature controlled animal unit where a photoperiod of 12 h light - 12 h darkness was maintained.

The temperature in the housing unit fluctuated between 17.3 °C and 24.9 °C. All cats were observed daily for general health and if required examined by a veterinarian.

The cats were fed daily with dry food at the recommended rate and water was available ad libitum and renewed at least twice daily.

### Allocation and treatment

Random allocation to the three study groups was performed within gender, based on individual pre-treatment flea counts. Group 1 was untreated while Groups 2 and 3 were treated topically with 0.5 ml of DF containing dinotefuran (22% *w*/w, 252.2 mg/ml) and fipronil (8.92% *w*/w, 98.9 mg/ml) on study day (day) 0 (Table 3). Actual doses administered to each cat in Group 2 ranged between 34.4 mg/kg and 49.8 mg/kg for dinotefuran, and between 13.5 mg/kg and 19.5 mg/kg for fipronil. Actual doses administered to each cat in Group 3 ranged between 27.2 mg/kg and 48.1 mg/kg for dinotefuran, and between 10.7 mg/kg and 18.9 mg/kg for fipronil. Using a 1 ml syringe, DF was applied topically. The hair was parted at the base of the neck in front of the shoulder blades, until the skin was visible. The content of the syringe was then administered directly on the skin at a single spot. The cats were observed for possible adverse events hourly for 4 h after the last animal had been treated. Local tolerance observations were conducted prior to treatment and at 4 h, 8 h, 1 day, 2 days and 3 days post-treatment.

**Table 3:** Details on flea retention as evaluated on day −5, as well as Investigational Veterinary Product (IVP) treatment dose (ml/kg) administered on day 0

Group	Treatment	Flea count (number per cat)			Treatment (ml/kg)		
		Mean	Min	Max	Mean	Min	Max
1	Untreated	65	60	73	–	–	–
2	DF	64	60	70	0.16	0.14	0.20
3	DF	66	60	74	0.14	0.11	0.19

*Abbreviations*: Min, minimum; Max, maximum; DF, treated with combination of dinotefuran and fipronil

### Flea infestation and counts

Laboratory reared fleas, originally isolated in the USA, were used for both in vitro and in vivo artificial infestations. At each infestation, every cat was infested with approximately 100 unfed mixed gender fleas.

**Fig. 1:**
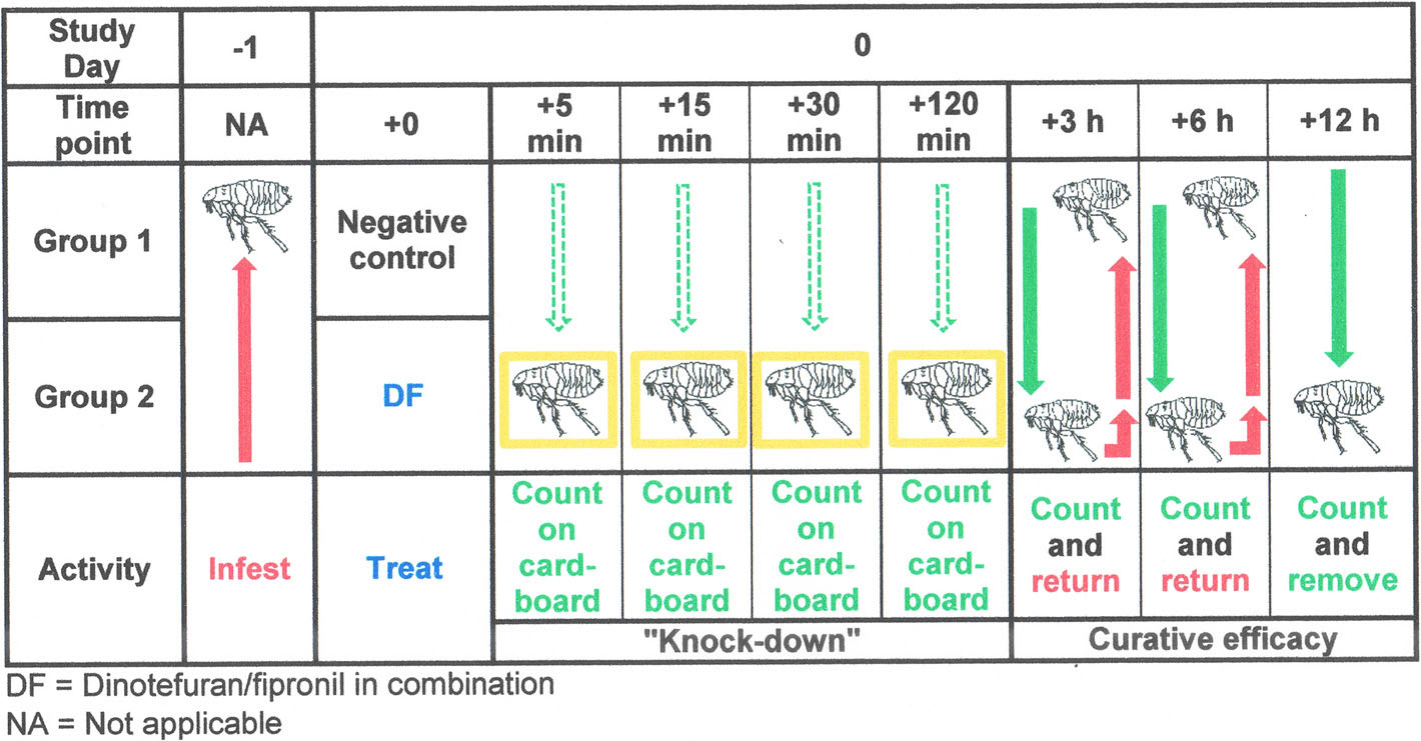
Flow diagram summarizing experimental design. Knock down and curative efficacy flea count assessments

**Fig. 2:**
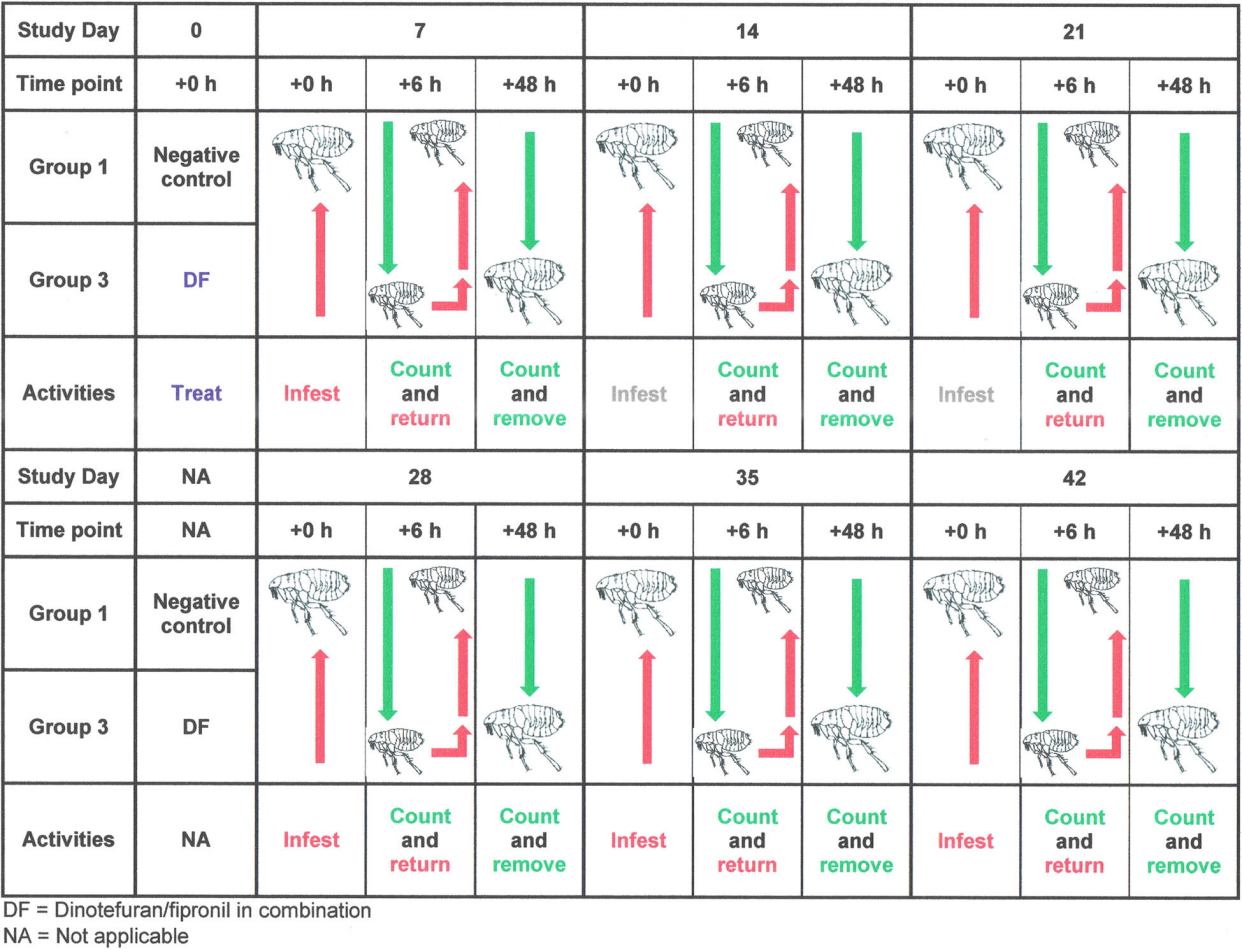
Flow diagram summarizing experimental design. Preventive efficacy flea count assessments

During acclimatization, all enrolled cats were infested with fleas on day -6 and the fleas were removed and counted on day -5, to determine the cat’s suitability for inclusion (Table 3). These counts were also used for subsequent group allocation purposes. Following allocation to the three study groups, flea infestations were performed on days -1, 7, 14, 21, 28, 35 and 42 for the control Group 1, only on the day prior to day 0 treatment (i.e. on day -1) for the DF treated Group 2, and only on the post-treatment days (i.e. on days 7, 14, 21, 28, 35 and 42) for the DF treated Group 3 (Figs. 1 and 2).

Flea counts were conducted by combing. Counts were performed on day 0 in Groups 1 and 2 at 3 h, 6 h and 12 h after treatment. On days 7, 14, 21, 28, 35 and 42, counts were performed at 6 h and 48 h after infestation in Groups 1 and 3. At the 3 h and 6 h time points, the fleas were returned to the animals, while they were removed at the final 12 h or 48 h assessment time points (Figs. 1 and 2).

Additionally, all fleas dropping from cats in Groups 1 and 2 were collected in collection pans placed underneath the cages after 5, 15, 30 and 120 min post-treatment, and counted (Fig. 1).

### Calculations and statistics

#### In vitro insecticidal activity and interaction between dinotefuran and fipronil

The mortality data derived from at least 2 independent replicates of duplicate testing, were analyzed using the CalcuSyn Version 2.0 software (Biosoft) and Sigmaplot version 12.5 (Systat software). Half maximal effective concentration (EC_50_) and combination index (CI) values were calculated to assess the potential for synergistic activity [29]. The CI was computed by CalcuSyn according to Chou [29]:

$$ CI=\frac{EC_{50}\kern0.5em  Dinotefuran\kern0.5em  in\kern0.5em  combination}{EC_{50}\kern0.5em  Dinotefuran\kern0.5em  alone}+\frac{EC_{50}\kern0.5em  Fipronil\kern0.5em  in\kern0.5em  combination}{EC_{50}\kern0.5em  Fipronil\kern0.5em  alone} $$

A CI value of approximately 1 indicated that the efficacy of the compounds was simply additive, a CI < 1 was interpreted as synergistic and a CI > 1 as antagonistic.

#### In vivo efficacy study in cats

Efficacies were calculated by comparing control *vs* DF treated flea counts using Abbot’s formula.

Efficacy values were calculated using geometric (GM) and arithmetic mean (AM) flea counts. For GM, the calculations were based on the flea (count +1) data, and one (1) was subsequently subtracted from the result to obtain a meaningful value for the geometric mean of the study groups. Groups were compared using a one-way ANOVA.

The proportion of dead/moribund/live dislodged fleas collected at each time collection point was calculated as follows:

Cumulative falling-off (%) = $\frac{\left(\frac{N_t}{T_t}-\frac{N_c}{T_c}\right)}{\left(1-\frac{N_c}{T_c}\right)}\times 100$

where N_t_ is the mean of the cumulative total number of dead/moribund/live fleas in the treated group (Group 2); N_c_ is the mean of the cumulative total number of dead/moribund/live fleas in the untreated control group (Group 1); T_t_ is the mean number of fleas infested to cats in the treated group (Group 2) = 100; and T_c_ is the mean number of fleas infested to cats in the untreated control group (group 1) = 100.

## Results

### In vitro insecticidal activity and interaction between dinotefuran and fipronil

No significant mortality was observed in the control treatments (solvent only and untreated vials), consequently mortality correction was not required to calculate EC_50_ values. Activity against *C. felis* was dose-dependent for both chemicals (Table 4). Dinotefuran (EC_50_ = 2.74 ppm) was more potent than fipronil (EC_50_ = 10.8 ppm) against fleas.

**Table 4:** EC_50_ values and combination indexes of single drugs and drug combination - *Ctenocephalides felis*, coated glass assay

Drug or drug combinations	EC_50_ (ppm)			Combination index (CI)
	Dinotefuran	Fipronil	Total amount of actives - Mean (95% confidence interval)	
Dinotefuran	2.74	–	2.74 (1.95–3.53)	–
Fipronil	–	10.80	10.80 (10.44–11.16)	–
Dinotefuran + fipronil 2:1	1.08	0.54	1.63 (1.53–1.73)	0.44

Tests to determine the effect of dinotefuran and fipronil in combination on adult fleas were conducted using a fixed ratio design [29]. Given that the efficacy against adult fleas of each compound separately was dose-dependent, the results were progressed into a synergy analysis. This analysis revealed that the combination was more effective against fleas than either compound alone, with a CI (combination index) of 0.44, indicating strong synergy (Table 4). Combination of dinotefuran with fipronil significantly shifted the dose response curve towards the left and significantly reduced the IC_50_ values of dinotefuran and fipronil, as indicated by the non-overlap of 95% confidence intervals (Table 4).

### In vivo efficacy study on cats

Topical DF administration was well-tolerated. Except for wet hair (cosmetic effects), no abnormal signs that could be attributed to treatment were observed. Crusts, associated with flea combing or excessive grooming of the cats were noticed on some animals, especially in the control group (4 out of 6), and were detected prior to treatment in some cats allocated to treated groups (1 and 2 out of 6, respectively). Flea-bite dermatitis was detected in one cat from the control group.

The flea retention rate on day -6 was > 60% (65 ± 5%). Moreover, at all post-treatment assessment time points the GM flea counts of the untreated control group were > 55.5 (AM > 49.5), indicating a vigorous flea challenge at each occasion (Tables 5, 6).

**Table 5:** The curative efficacy of a combination of dinotefuran/fipronil administered topically on Day 0 following infestation of cats with *Ctenocephalides felis* on Day -1

Day 0 time point	Variable	Flea counts	
		Geometric mean	Arithmetic mean
3 h	Untreated (Group 1)	69.2	71.0
	DF (Group 2)	1.8	2.0
	Percentage efficacy	97.4	97.2
6 h	Untreated (Group 1)	60.5	62.8
	DF (Group 2)	0	0
	Percentage efficacy	100	100
12 h	Untreated (Group 1)	57.8	59.6
	DF (Group 2)	0.1	0.1
	Percentage efficacy	99.8	99.8

*Abbreviation*: DF, dinotefuran/fipronil combination

**Table 6:** Preventative efficacy of dinotefuran/fipronil administered topically to cats on day 0, against re-infestation with *Ctenocephalides felis* on days 7, 14, 21, 28, 35 and 42

Time point	Criteria	Mean flea counts on day (D)											
		D7		D14		D21		D28		D35		D42	
		GM	AM	GM	AM	GM	AM	GM	AM	GM	AM	GM	AM
6 h	Untreated	62.7	64.3	60.2	62.1	68.9	70.4	75.1	76.4	70.2	72.0	71.9	73.6
	DF	0.6	0.9	0.5	0.8	1.9	2.8	4.3	7.0	15.2	23.9	26.6	33.6
	Efficacy (%)	99.1	98.6	99.2	98.8	97.3	96.1	94.2	90.8	78.3	66.8	63.0	54.3
48 h	Untreated	57.8	58.4	48.8	49.5	65.9	67.9	48.1	50.4	55.2	58.6	54.5	55.8
	DF	0.0	0.0	0.1	0.3	0.4	0.9	0.1	0.1	1.4	3.0	2.6	3.9
	Efficacy (%)	100	100	99.7	99.5	99.4	98.7	99.8	99.8	97.4	94.9	95.2	93.0

*Abbreviations*: GM, geometric mean; AM, arithmetic mean; DF, dinotefuran/fipronil combination

### Onset of activity and curative efficacy (Group 1 *vs* Group 2)

Significantly fewer fleas (Table 7) were recorded on cats in the DF treated group (Group 2) than on cats in the untreated control group (Group 1) at all post-treatment assessment time points (3 h, 6 h and 12 h).

**Table 7:** Results of ANOVA comparisons between groups

Variable	Time point	Study day	ANOVA result	
			Arithmetic mean	Geometric mean
Curative efficacy (Group 1 *vs* Group 2)	3 h	0	*F*_(1,14)_ = 118.08, *P* < 0.0001	*F*_(1,14)_ = 371.28, *P* < 0.0001
	6 h		*F*_(1,14)_ = 101.36, *P* < 0.0001	*F*_(1,14)_ = 1553.43, *P* < 0.0001
	12 h		*F*_(1,14)_ = 115.48, *P* < 0.0001	*F*_(1,14)_ = 964.38, *P* < 0.0001
Preventative efficacy (Group 1 *vs* Group 3)	6 h	7	*F*_(1,14)_ = 148.72, *P* < 0.0001	*F*_(1,14)_ = 279.06, *P* < 0.0001
		14	*F*_(1,14)_ = 111.47, *P* < 0.0001	*F*_(1,14)_ = 269.66, *P* < 0.0001
		21	*F*_(1,14)_ = 142.48, *P* < 0.0001	*F*_(1,14)_ = 128.82, *P* < 0.0001
		28	*F*_(1,14)_ = 149.25, *P* < 0.0001	*F*_(1,14)_ = 44.38, *P* < 0.0001
		35	*F*_(1,14)_ = 26.42, *P* = 0.0002	*F*_(1,14)_ = 13.08, *P* = 0.0028
		42	*F*_(1,14)_ = 19.45, *P* = 0.0006	*F*_(1,14)_ = 9.85, *P* = 0.0073
	48 h	9	*F*_(1,14)_ = 353.47, *P* < 0.0001	*F*_(1,14)_ = 6013.11, *P* < 0.0001
		16	*F*_(1,14)_ = 236.02, *P* < 0.0001	*F*_(1,14)_ = 621.06, *P* < 0.0001
		23	*F*_(1,14)_ = 109.50, *P* < 0.0001	*F*_(1,14)_ = 216.72, *P* < 0.0001
		30	*F*_(1,14)_ = 75.43, *P* < 0.0001	*F*_(1,14)_ = 702.01, *P* < 0.0001
		37	*F*_(1,14)_ = 49.96, *P* < 0.0001	*F*_(1,14)_ = 61.03, *P* < 0.0001
		44	*F*_(1,14)_ = 109.33, *P* < 0.0001	*F*_(1,14)_ = 78.76, *P* < 0.0001

*Abbreviations*: ANOVA, analysis of variance; Time point, time in hours after administration of the Investigational Veterinary Product (curative efficacy), or time in hours after flea infestation (preventative efficacy); h, hours

The efficacy of DF against an existing population of fleas was 97.4% (97.2%, AM) at 3 h after treatment and at 12 h it had increased to ≥ 99.8% (GM and AM) (Table 5).

### Preventative efficacy (Group 1 *vs* Group 3)

Significantly fewer fleas (Table 7) were recorded on cats in the treated group (Group 3) than on cats in the untreated control group (Group 1) at all post-treatment assessment time points and days.

At 6 h after re-infestations, the efficacy of DF remained ≥ 90.8% (GM and AM) for up to 28 days and efficacy was maintained at ≥ 54.3% (GM and AM) for up to 42 days post-treatment (Table 6). At 48 h after re-infestations, DF remained almost fully effective for up to 28 days with efficacies ≥ 99.4% (GM and AM) and remained ≥ 93.0% (GM and AM) effective for up to 42 days post treatment (Table 6).

### Knock-down effect (Group 1 *vs* Group 2)

**Fig. 3:**
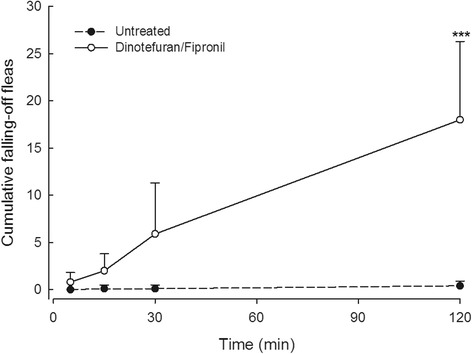
Cumulative number of fleas (arithmetic mean + standard deviation) that dropped from cats in Group 1 (untreated animals) and Group 2 (dinotefuran/fipronil treated animals)

Very few fleas (mean ≤ 1.0) were collected from the pans placed under the control cats, indicating vigorous on-host infestations. In contrast, the cumulative number of fleas dislodged and collected from the treated cats increased gradually from 5 min after treatment, and was greater at 2 h post-treatment, compared to the number collected from control cats (Fig. 3).

## Discussion

The successful protection of cats against the detrimental effects of flea infestations, which may include FAD and possibly also flea borne diseases, is dependent on the use of a pulicidal product with rapid and persistently maintained activity. To achieve these performances, the combination of active ingredients with complementary modes of action and potencies in a single spot-on formulation has been developed. Dinotefuran, a third generation neonicotinoid and fipronil, a phenylpyrazole were selected. As a first step, the insecticidal efficacy of the two actives separately and in combination were tested against *C. felis* in vitro. This test indicated that dinotefuran was more potent than fipronil, and when they were administered in a 2:1 combination, a strong synergistic effect was observed in vitro. It was assumed that this synergistic effect would enhance not only the in vivo duration of activity of the combination against *C. felis*, but would also promote and maintain a quick speed of kill over this period. In the subsequent in vivo study, the persistent efficacy of DF remained ≥ 95.2% (GM counts) for 42 days. This is 12 days longer than previously reported for dinotefuran in combination with an insect growth regulator without adulticidal activity (pyriproxyfen) [22, 30]. Not only did the duration of efficacy improve, but the dose volume of a 22% *w*/w (252.2 mg/ml) DF formulation decreased to 0.5 ml/cat, where it was previously applied at 0.8 ml on cats that weighed between 2.4 kg and 3.9 kg [22]. In the current study, the dose rate of dinotefuran administered to individual animals thus ranged between 27.2 mg/kg and 49.8 mg/kg, compared to a range of between 51.7 mg/kg and 84.1 mg/kg administered by Murphy et al. [22]. Moreover, the speed of kill of the combination was also markedly improved in comparison to fipronil-based formulations [31]. In the latter study, the authors demonstrated that a fipronil/(s)-methoprene formulation administered to cats at a dose of 7.5 to 15 mg/kg was only 41.1% effective against infestation with a Kansas 1 (KS1) *C. felis* strain at 6 h post-infestation, assessed 28 days post-treatment [31]. The efficacy demonstrated in that study was markedly lower than that observed for the DF formulation in the current study, where a 94.2% efficacy (GM) was observed at the same assessment time point and remained ≥ 63.0% up to 42 days post-treatment. Such difference can be explained not only by the flea strain differences, but also by an improved activity related to the synergy between the two active ingredients tested in the present study. The rapid and maintained action of DF is advantageous for protecting cats against the detrimental health effects of flea infestations. By treating cats with DF, they can rapidly be rid of fleas even when repeatedly exposed under high environmental flea challenges. This will aid in preventing or rapidly relieving cats suffering from dermatological symptoms associated with severe flea infestations. Moreover, the rapid and maintained action of DF can assist in the protection against flea-borne pathogens. For instance, *Bartonella henselae*, a Gram negative bacteria responsible for a zoonotic disease, is excreted in flea feces within 24 h after a blood meal [32] and fleas can start to excrete faeces about 30 min after infestation. Consequently, killing fleas as fast as possible before they excrete infective faeces, will contribute to the protection of cats and their owners against infection.

The combination of actives with different modes of activity can be beneficial in controlling infestations with specific field isolates that may be less susceptible to a specific active ingredient. For example, fipronil has a modest killing effect on the KS1 flea strain while dinotefuran is highly potent [21, 33]. The combination of these actives can therefore reduce the risk of potential efficacy failures in the field due to the presence of less susceptible flea populations.

An obvious advantage of the DF formulation is the well-known acaricidal activity of fipronil, affecting not only the central nervous system of the parasite, but also important organs of ticks such as the saliva glands and ovaries. This attribute aids in preventing disease transmission as well as parasite reproduction [18, 34].

Although the experimental DF formulation used in this study exhibited great benefits and promise, it can potentially be improved by including a potent insect growth regulator (IGR). This will not only result in the on host-control of fleas, but the effective control of fleas in the animal’s immediate environment. Pyriproxyfen, a potent juvenile hormone analog effectively inhibiting the development of fleas in the environment, would be the appropriate choice. This molecule prevents eggs from hatching, and the developmental stages from molting and ultimately checks *C. felis* proliferation in the environment [35, 36].

## Conclusions

The combination of dinotefuran and fipronil in a single formulation exhibited strong synergistic insecticidal activity against *C. felis* as assessed in vitro. This translated into a rapid and maintained insecticidal activity against *C. felis* infestations on cats (in vivo). The rapid curative insecticidal effect was observed as early as 3 h after treatment and as early as 6 h against re-infestations for up to 6 weeks post-treatment. Because of its insecticidal activity profile, DF can be considered as a reliable ectoparasiticide combination to protect cats against flea infestations and associate diseases.

## Abbreviations

AM: Arithmetic mean
GM: Geometric mean
